# P-1797. The Effect of Recipient Age and Cytomegalovirus Sero-status on Patient and Graft Survival in Liver Transplant Recipients

**DOI:** 10.1093/ofid/ofaf695.1966

**Published:** 2026-01-11

**Authors:** Abhay Dhand, Seigo Nishida, Kenji Okumura

**Affiliations:** Westchester Medical Center, Valhalla, NY; Westchester Medical Center, Valhalla, NY, Valhalla, New York; Westchester Medical Center, Valhalla, NY, Valhalla, New York

## Abstract

**Background:**

Cytomegalovirus (CMV) may impact allograft function and overall survival in solid organ transplant recipients in various direct or indirect mechanisms. The association between recipient age and donor-recipient CMV status and outcomes of liver transplantation (LT) are unclear.

**Figure:**
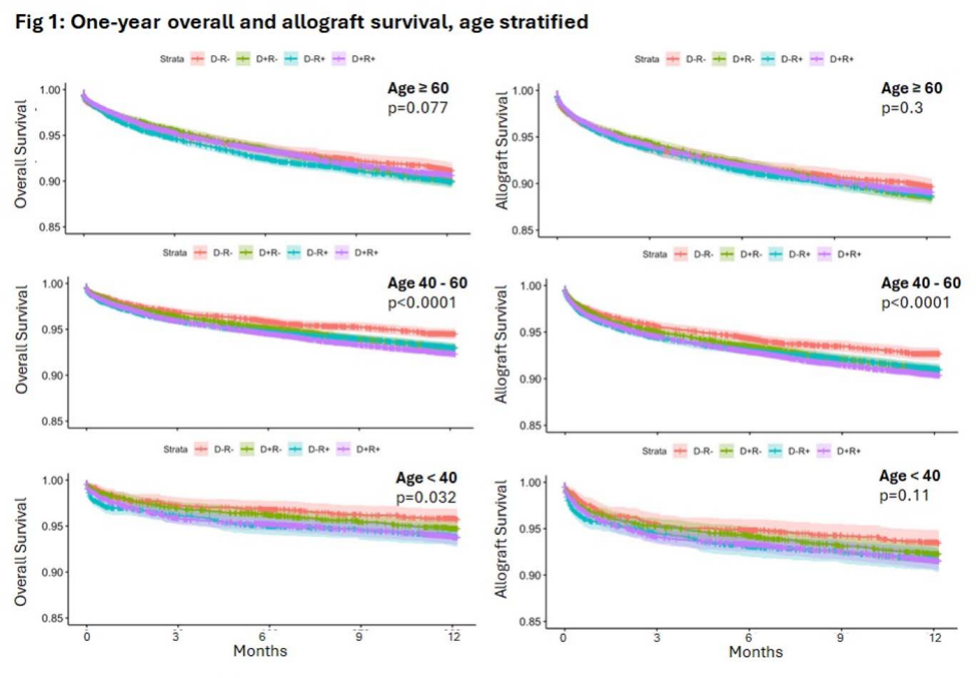

**Figure:**
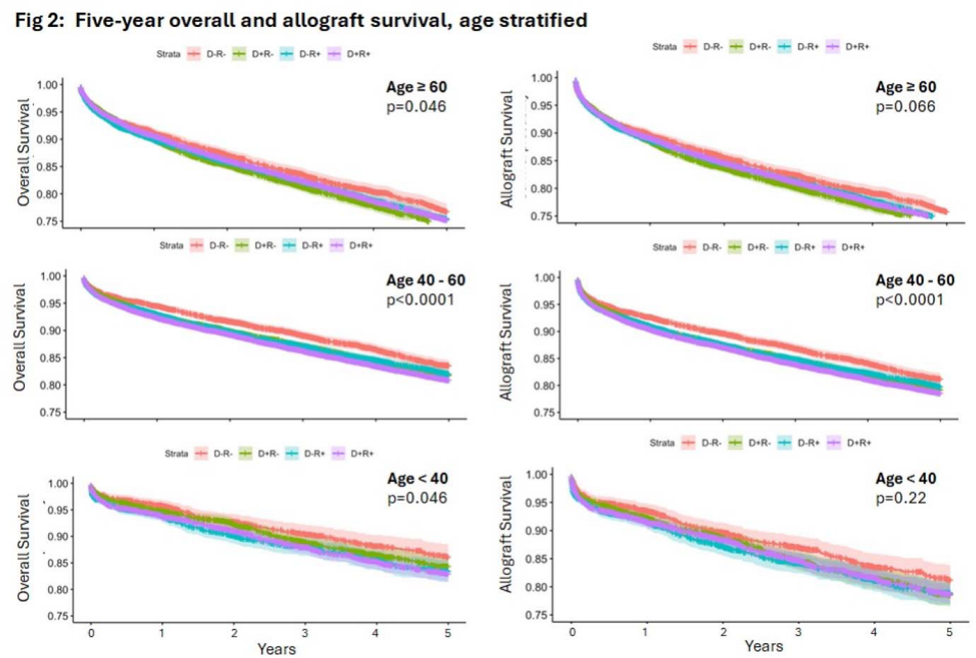

**Methods:**

Retrospective analysis of the Scientific Registry of Transplant Recipients for LT recipients ≥18 years old between 2000-2020 (N=89,753) was conducted. Recipient age-based stratification analysis was performed to compare the association of CMV serostatus and recipient age on overall and graft survival after LT. Survival curves were generated using the Kaplan-Meier method. All-cause mortality and graft failure hazard ratios were calculated at one- and five-years post-LT.

**Figure:**
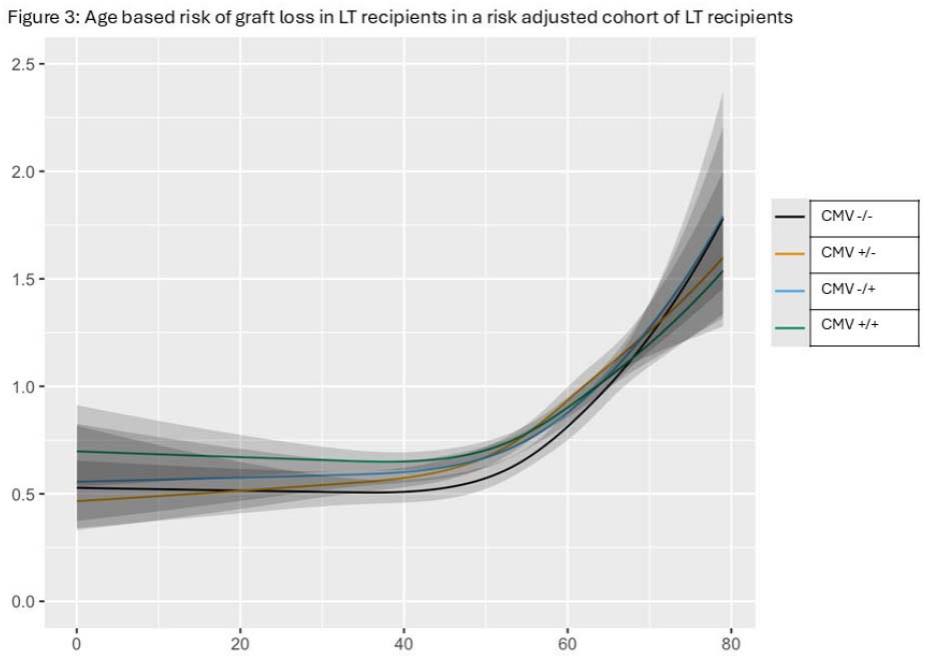

**Results:**

LT recipients were stratified in to age groups (years) of ≥ 60 (36,636; 40.8%), 40-60 (43,921; 48.9%), and < 40 (9,196; 10.3%). Among these LT recipients, various CMV serostatus groups were D-R- (12,521;13.8%), D+R- (20,815; 23.0%), D-R+ (19.830; 21.9%), and D+R+ (37,395; 41.3%). In the age-based stratification analysis, all CMV seropositive statuses (D+R-, D-R+, D+R+) when compared to CMV D-/R-, were negatively associated with overall and graft survival at both one- and five-years post-LT (p< 0.0001), group. The negative association of CMV seropositivity on one-year and five-year overall and graft survival was most pronounced in 40-60 years group (Fig 1, 2) when compared to the other two age groups.

**Conclusion:**

During the current era of effective CMV prophylaxis, LT recipients in the 40-60-year age group are at the highest risk of CMV seropositivity (D+R-, D-R+, D+R+) associated worse overall and graft survival at both one- and five-years post-LT. Identification of age-based risk factors, increased awareness, as well as effective diagnostic and therapeutic strategies are needed to mitigate this risk.

**Disclosures:**

Abhay Dhand, MD, Eurofins Viracor: Advisor/Consultant|Eurofins Viracor: Honoraria|Merck: Advisor/Consultant|Merck: Honoraria

